# Revolutionizing cancer treatment: The role of radiopharmaceuticals in modern cancer therapy

**DOI:** 10.1002/pro6.1239

**Published:** 2024-09-11

**Authors:** Treesa P. Varghese, Anish John, Jithin Mathew

**Affiliations:** ^1^ Department of Pharmacy Practice Yenepoya Pharmacy College & Research Centre, Yenepoya (Deemed to be University), Naringana Mangalore Karnataka India; ^2^ Department of Pharmaceutics NGSM Institute of Pharmaceutical Sciences Nitte (Deemed to be University), Deralakatte Mangalore Karnataka India

**Keywords:** radiopharmaceutical therapy, radiopharmaceuticals, quality control, scintigraphy

## Abstract

Radiopharmaceutical therapy (RPT) is a precision medicine approach that involves the targeted delivery of radioactive atoms to tumor cells, representing a breakthrough strategy for cancer treatment. Radiopharmaceuticals typically consist of a small amount of radioactive material, a radionuclide, paired with a chemical that specifically targets the cell. Some radionuclides naturally target specific cells or biological processes without the need for modification. RPT is a novel cancer treatment method that offers various advantages over current traditional treatment approaches. One of the primary advantages of RPT is its ability to target cancer cells, including those in metastatic areas. Another key advantage of RPT is that radiation can be delivered systemically, locally, or physiologically to specific cells internally rather than being applied externally. Moreover, radiotracer imaging can be utilized to determine radiopharmaceutical absorption in target tissues before providing a therapeutic dose. Compared to all other cancer treatment approaches, RPT has demonstrated high efficacy with minimal toxicity. The recent approval of multiple RPT medicines by the US Food and Drug Administration highlights the tremendous potential of this treatment. This article provides a detailed review of RPT, including insights into manufacturing procedures, safety measures, and its applications in cancer therapy.

## Graphical abstract

Steps involved in the preparation of radiopharmaceuticals

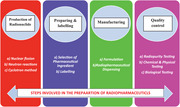

## 1 INTRODUCTION

Radiopharmaceutical therapy (RPT) involves the targeted delivery of radiation directly to tumor cells or the tumor microenvironment using a radioactive drug. In this approach, radioactive atoms are administered systemically to target cancer cells or their microenvironment. RPT represents a novel strategy in cancer treatment, offering significant advantages over traditional approaches like external beam radiotherapy and brachytherapy. RPT is also known as radioligand therapy, theranostics, or molecular radiotherapy. It involves the use of medications that exhibit greater selectivity in binding to cancer cells or accumulate through physiological processes.^1^ The use of radioactive chemicals in diagnosis and treatment is known as radiopharmaceuticals, which can be administered as a basic salt or combined with more intricate compounds.^2^ RPT is an innovative method characterized by minimal toxicity with great potential for effectiveness in cancer management. One of its primary benefits is the ability to target cancers, even in metastatic locations.^3^ Before administering a therapeutic dosage, radiopharmaceuticals can be employed in radiotracer imaging to assess their absorption in target tissues. Additionally, a broad spectrum of commercially accessible radionuclides with diverse radiation types and intensities is now available. For example, high linear energy transfer (LET) radionuclides are effective in eliminating hypoxic cells resistant to oxygen. Furthermore, this treatment allows for a comparatively lower dose to be absorbed by the entire body.^4^

In contrast to conventional medications, radiopharmaceuticals typically have a short half‐life due to their rapid decay, requiring them to be prepared just before clinical use. This rapid decay means that comprehensive quality control (QC) of the finished product is not feasible; for example, there is insufficient time to conduct sterility testing. Therefore, it is essential that radiopharmaceuticals are prepared and administered safely and effectively to ensure the safety of the operators and patients. According to the recommendations of the Atomic Energy Regulatory Board of India, the use of radioactive materials necessitates careful handling and secure use by trained and authorized personnel within authorized laboratory facilities.^5^ This review aims to provide a comprehensive overview of the principles, manufacturing process, and therapeutic applications of radiopharmaceuticals in cancer therapy. It also aims to discuss safety concerns and the challenges in the production and development of these advanced therapeutic agents.

## 2 PRINCIPLE

Radiopharmaceuticals or radioactive drugs are substances that contain one or more radionuclides and are used in the diagnosis and treatment of diseases, including cancer. Radiopharmacy or Nuclear Pharmacy is a pharmacy specialty that focuses on the safe and effective use of radiopharmaceuticals.^6^ The physical and molecular properties of a radiopharmaceutical influence its distribution within the body, whereas its radioactive decay qualities determine how it is detected and its suitability for diagnostic or therapeutic purposes.^7^ Therapeutic radionuclides emit radiation that delivers a high dose to the specific tissue, whereas radiation from diagnostic radionuclides helps visualize the distribution of the labeled compound in the body. The specificity for localization should be considered before the therapeutic use of a radiopharmaceutical. To accomplish this localization, certain biological concepts such as active transport and binding to cellular constituents have been applied.^8^

Radiopharmaceuticals are composed of a radioactive isotope attached to a targeting molecule, such as monoclonal antibodies, proteins, peptides, or small molecules, that binds specifically to cancer cells.  Precisely, radiopharmaceuticals exert anticancer effects by inducing cytotoxic DNA damage through various mechanisms, including the generation of reactive oxygen species, induction of single and double‐strand breaks, and the inhibition of DNA repair processes. Upon administration, these compounds accumulate only in tumor cells.^9^, ^10^ The radioactive isotopes emit ionizing radiations in the form of alpha, beta, or gamma particles, which penetrate cancer cells and damage their DNA, resulting in cell death by apoptosis or necrosis. Furthermore, this radiation can alter the tumor environment, limit angiogenesis, and regulate the immune response, thus improving the overall therapeutic effect.^11^

## 3 PRODUCTION OF RADIOPHARMACEUTICALS

The production of radiopharmaceuticals carries potential hazards. Preparing radiopharmaceuticals for cancer therapy involves several complex steps, including radionuclide production, preparation, irradiation, and chemical processing, labeling of the active ingredient, manufacturing the desired dosage form, and conducting QC^12^.

### 3.1 Production of radioactive isotopes

Radionuclides are artificially produced through nuclear activation in a nuclear reactor. In this process, stable elements are subjected to excessive neutron bombardment within the reactor, leading to the formation of radionuclides when stable atoms undergo neutron capture. Radioactive isotopes or radionuclides can be produced through various methods, including nuclear fission, neutron reactions, and cyclotron methods.^13^

- ***Nuclear fission***: Nuclear fission involves splitting atomic nuclides into smaller nuclei. Some unstable nuclei undergo spontaneous fission, while others require additional energy to overcome nuclear binding forces. This energy is typically supplied by the absorption of neutrons.^14^
- ***Neutron reactions***: Radionuclides are generated through neutron activation or transmutation processes. In a nuclear reactor, specific materials are bombarded with neutrons, leading to the formation of several radioisotopes, such as ^131^I (used for thyroid cancer) and ^153^Sm (used for prostate cancer). Transmutation can produce radioactive phosphorus, which can be isolated by chemical methods.^15^
- ***Cyclotron method***: Cyclotrons are commonly employed to accelerate charged particles like ^90^Y.  In nuclear medicine, different cyclotrons are used to generate medical isotopes, particularly for cancer treatment, to deliver radiation. Medical cyclotrons are used globally to generate medical isotopes such as ^18^F and ^11^C. Other cyclotrons generate radiation beams for cancer therapy.^16^

### 3.2 Target material preparation and irradiation

The substance to be irradiated is called the target material, which should be carefully selected and prepared. This material may contain materials or compounds that, when exposed to neutrons or protons, convert into the appropriate radioactive isotope. The target material is subjected to a flux of neutrons in a reactor or protons in a cyclotron, resulting in nuclear processes that transform the stable isotopes within the target material into radioactive ones (Table 1).

**TABLE 1:** FDA‐approved radio pharmaceutical therapies for cancer management.^2^

RADIO NUCLEOTIDE	APPLICATION
Calcium Chloride [Ca‐45/Ca‐47]	Investigation into disorders related to calcium metabolism, bone cancer, and various abnormalities affecting the bones.
Cyanocolamine Co‐60	Co‐60 treatment of advantage stages of cancer affecting the cervix, vagina, uterus, blade and mouth, and tongue.
Sodium iodide [I‐131]	Treatment of thyroid cancer.
Samarium‐153	Pain relief for patients with bone metastases and treatment of prostate cancer.
Yttrium‐90 (Y‐90)	Used in radioembolization procedures for liver cancer (hepatocellular carcinoma) and in treating non‐Hodgkin lymphoma
Radium‐223 dichloride	Treatment of metastatic castration‐resistant prostate cancer with symptomatic bone metastases.
Lutetium‐177	Treats gastroenteropancreatic neuroendocrine tumors

### 3.3 Chemical processing and labeling of the ingredients

After irradiation, the target material undergoes chemical processes to isolate the desired radioactive isotope. Common chemical separation methods include solvent extraction, precipitation, and chromatography. Radioactive labeling involves substituting an atom in its stable isotope with another isotope of a similar element that is radioactive. This step is crucial to ensure that the radiopharmaceuticals will effectively target cancerous cells. Radiolabeling can be achieved through different methods such as incorporation of a foreign label, exchange of isotopes, and chelation.^17^, ^18^

### 3.4 Manufacturing the desired dosage form

The final dosage form of a radiopharmaceutical is prepared similarly to that of non‐radioactive medicines. This process involves pH adjustment, adding stabilizers, and ensuring the solution is sterile and pyrogen‐free.^19^ Common dosage forms include tablets, capsules, injectable solutions, and radioactive seeds or implants. For instance, ^131^I is readily available in capsule form for treating hyperthyroidism and certain types of thyroid cancer. Iodine‐125 radioactive seeds are used in prostate cancer treatment. Lutetium‐177 in the form of injectable solutions is widely used for treating neuroendocrine tumors (NETs).^20^

### 3.5 Quality control

QC testing for radiopharmaceuticals is critical in safeguarding patient safety, ensuring efficacy, maintaining regulatory compliance, achieving consistency, and fostering public confidence in these vital medical products.^21^ Since radiopharmaceuticals are administered to patients for diagnostic imaging or therapeutic purposes, rigorous testing helps minimize the risk of adverse reactions and ensures accurate diagnostic results or therapeutic outcomes.^19^ Regulatory agencies mandate QC testing to ensure compliance with established standards and regulations, with failure to meet these requirements potentially leading to regulatory penalties or product recalls. QC testing for radiopharmaceuticals encompasses a comprehensive evaluation of various products and materials involved in their production.^22^ Raw materials, including precursor chemicals, reagents, and radioactive isotopes, undergo testing for purity, identity, and suitability for use in the manufacturing process. Intermediate products undergo rigorous testing to meet specifications before further processing.

Finished radiopharmaceuticals undergo extensive QC testing, including identity, purity, sterility, endotoxin levels, stability, and potency assessment. This ensures that the products adhere to strict quality standards, enhancing safety, efficacy, and reliability for patient use.^23^ Various methods are employed in QC testing for radiopharmaceuticals to ensure safety, efficacy, and quality. High‐performance liquid chromatography and thin‐layer chromatography are used to analyze purity, with acceptable thresholds typically set at over 95% based on the particular radiopharmaceuticals. Gamma spectroscopy is used to confirm the correct radionuclide and ensure it meets specific radioactivity levels.  The product must be free of microbiological contamination, and sterility is evaluated using techniques like membrane filtering or direct inoculation. The limulus amebocyte lysate test is used to measure endotoxin levels, with pharmacopeial standards determining acceptable limits (e.g., <175 EU per dose for intravenous products). Stability testing involves evaluating the shelf‐life and degradation of products over time to ensure that the radiopharmaceutical remains within its predetermined potency and purity limits until its expiration date. Potency is determined by measuring radioactivity concentration, which must match the labeled amount within a given range, usually ±10%.^24^, ^25^

Advanced testing methods such as microfluidic QC systems can effectively reduce the sample, reagent consumption, and time of testing, which are very crucial in radiopharmaceutical testing.^26^ These quality testing methods and standard guidelines are established by regulatory agencies such as the US Food and Drug Administration (FDA), European Medicines Agency (EMA), and International Atomic Energy Agency (IAEA). Each of these agencies has its unique regulations governing radiopharmaceuticals alongside shared regulatory frameworks. These regulatory authorities govern the licensing of production, use, storage, transportation, and dispensing of radioactive materials.^27^ Manufacturers must comply with Good Manufacturing Practice (GMP) guidelines for the production, QC, transportation, and utilization of radiopharmaceuticals. These regulations ensure that radiopharmaceuticals are safe and meet standards for identity, potency, quality, and purity. Noncompliance with these manufacturing, QC, and handling standards of radiopharmaceuticals poses risks to patient safety, regulatory compliance, and the manufacturer's reputation. Stringent regulations are essential for ensuring product safety and maintaining public trust.^28^

### 3.6 Packaging and distribution

**FIGURE 1:**
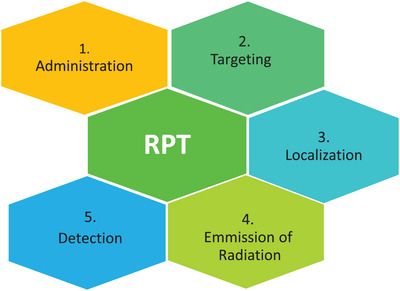
Diagram represents the action of radiopharmaceuticals from administration to site of action.

After completing the formulation and QC steps, radiopharmaceuticals must be carefully packaged to ensure sterility, stability, and safety. These products should be packaged in specialized containers, which are generally lead‐lined or constructed of other radiation‐shielding materials, to protect the operators and patients from radiation exposure. In addition, the packaging must be robust and securely sealed to prevent leaks or contamination. Furthermore, temperature control is critical for some radiopharmaceuticals, necessitating insulated packaging to maintain appropriate temperature conditions during transport. Regulatory compliance is an important part of packaging since radiopharmaceuticals are governed by strict regulations from organizations such as the FDA, the Nuclear Regulatory Commission, and their international equivalents.^29^ These standards require specific labeling, such as the radioactive symbol, product information, activity level, and handling instructions, to ensure proper recognition and safety. The half‐life of the radioisotopes must be considered when planning distribution operations, as timely delivery is critical to maintaining efficacy. This often requires prompt transportation, sometimes within hours, from manufacturing sites to healthcare professionals. Upon arrival at their destination, typically a healthcare facility or institution, trained professionals inspect the shipment for integrity and documentation before storage or use. The ultimate purpose of the packaging and distribution process is to ensure that radiopharmaceuticals reach patients in optimal condition, reducing radioactive exposure to handlers and the environment while maintaining the product's therapeutic efficacy (Figure 1).^30^

## 4 SELECTION CRITERIA FOR RADIOPHARMACEUTICALS

Selecting an appropriate radionuclide for therapeutic applications involves considering various factors, including the radionuclide's physical properties, particularly the type and energy of radiation it emits, its half‐life, and the precise location of the target area to be treated. The deposition of energy is measured using LET, which differs for alpha, beta, or gamma radiation. Beta‐emitting radionuclides are often used for therapeutic purposes.^31^ In therapeutic nuclear medicine, only radionuclides that emit beta particles or absorb electrons and release Auger electrons have been used. Beta particles can penetrate tissue to depths of a few centimeters to a few millimeters, which is suitable for irradiating small‐ to medium‐sized tumors. Some of the most promising beta‐emitting radionuclides possess appropriate half‐lives, ranging from several hours to days. Additionally, the production of several of these radionuclides is relatively straightforward in nuclear research reactors, facilitating their availability.^32^

## 5 RADIOPHARMACEUTICALS FOR CANCER THERAPY

Current cancer treatments often involve a combination of surgery, radiation therapy, and chemotherapy. Recent advances in the understanding of the tumor microenvironment have led to the development of new cancer therapies. Cancer tissue comprises cellular and non‐cellular components, such as vascular and interstitial elements, which differ significantly from the surrounding normal tissue. These differences pose challenges for the local delivery of drugs to tumor cells. Radiopharmaceuticals have emerged as effective agents for both diagnostics and therapy due to their ability to target tumors precisely.^33^

### 5.1 Targeted tumor therapy

Targeted tumor therapy using radiopharmaceuticals is an innovative approach to cancer treatment that aims to deliver large amounts of radiation directly to cancer cells while minimizing damage to healthy tissue. This therapy utilizes radiopharmaceuticals that selectively bind to tumor cells and deliver a dose of radiation that is lethal to the cancer cells. An example of radiopharmaceutical used in targeted tumor therapy is ^131^I‐labeled antibodies, which are effective in the treatment of lymphomas and other types of cancers. Targeted tumor therapy using radiopharmaceuticals is an exciting area of research that has the potential to revolutionize cancer treatment. The use of radiopharmaceuticals that selectively bind to tumor cells and deliver a high dose of radiation has shown promising results in the treatment of various types of cancers. Ongoing research in this field is focused on developing new radiopharmaceuticals that can target specific types of cancer cells with even greater precision, further improving the effectiveness of targeted tumor therapy.^34^

### 5.2 Radionuclide delivery systems and organic nanoparticles in nuclear medicine for cancer therapy

The success of radionuclide applications in pre‐clinical and clinical studies largely depends on selecting an appropriate delivery platform.^35^ Various factors, including the type and size of the targeted cancer, the density and heterogeneity of the target, and the LET, influence the choice of radionuclide. β‐ emitters, characterized by a low LET (0.2 keV/mm) and a relatively long penetrating range in tissue (one to several mm), can penetrate deeply into tumors but may also partially damage surrounding normal tissues. Common β‐ emitters like ^177^Lu, ^90^Y, and ^131^I have been utilized in thyroid cancer treatment, with both iodine and lutetium isotopes emitting a detectable γ photon for single‐photon emission computed tomography imaging.^36^ Radionuclides undergoing β+ decay emit positrons and are primarily used for diagnostic purposes, detectable via positron emission tomography. Short‐lived β+ emitters such as ^15^O, ^13^N, ^11^C, and ^18^F are commonly employed to locate diseases and quantitatively assess biochemical and physiological processes. However, there is growing interest in long‐lived β+ emitters like ^89^Zr, ^64^Cu, and ^52^Mn, as they allow for extended monitoring of treatments over 2–3 weeks.^37^

Drug delivery systems are essential in enhancing the concentration of drugs at the target site and improving pharmacokinetic profiles. Targeted drug delivery typically involves active or passive targeting mechanisms, which also applies to radionuclide delivery.^38^ Utilizing delivery systems that ensure precise delivery of the radionuclide cargo is essential for both therapeutic and diagnostic radionuclides. Improved delivery of radionuclides can reduce the required dosage per patient, thus lowering the risk of exposure and associated costs.^39^

Organic nanoparticle (NP) systems are extensively utilized in nuclear medicine for delivering radionuclides, focusing on their design considerations, recent advancements, modifications, and radiolabeling techniques. These NPs are efficient carriers for various biologically active substances, including radionuclides.^40^ Currently, a wide range of organic NPs have been developed and categorized into five main types used in nuclear medicine for both therapy and diagnostics, including liposomes, albumin‐based NPs, dendrimers, polymeric micelles, and polymeric NPs. Numerous techniques have been developed for radionuclide labeling and surface modification of these organic NPs using targeting ligands, enabling precise delivery of radionuclides to specific sites, such as tumor areas.^41^

### 5.3 Peptide receptor radionuclide therapy

Targeted radionuclide therapy, such as peptide receptor radionuclide therapy (PRRT), uses systemic delivery of therapeutic peptides labeled with radionuclides that specifically target NETs. This approach allows the direct delivery of radiation to the malignant tissue while sparing healthy cells.^42^ Typically, a radiopharmaceutical consists of a peptide and a radioactive isotope, like ^90^Y or ^177^Lu, which release radiation that kills tumor cells. PRRT targets somatostatin receptors, which are over‐expressed on the surface of NET cells, helping to control tumor growth and improve quality of life. Current investigations aim to improve therapeutic outcomes by refining treatment procedures, enhancing patient selection criteria, and investigating new radionuclides and targeting peptides.^43^

### 5.4 Palliative care

RPT can effectively alleviate metastatic bone pain. Many clinical studies have demonstrated the efficacy and safety of RPT in managing pain associated with bone metastasis. The treatment involves using radioactive substances that target and destroy cancerous cells within the bone.^43^ These radiopharmaceuticals are administered via injection or orally, and once they enter the bloodstream, they bind to the cancerous cells in the bone, emitting radiation that destroys them. This therapy is effective in reducing pain and improving the quality of life in patients with metastatic bone pain. Some commonly used radiopharmaceuticals include ^89^Sr, ^153^Sm, and ^223^Ra.^44^

### 5.5 Targeted alpha therapy

Targeted alpha therapy (TAT) is an innovative approach in palliative care that can be used for a wide range of tumor types. Pre‐clinical and clinical research data demonstrate the potent cytotoxicity of alpha particles to target cancer cells while sparing surrounding healthy tissues.^45^ TAT uses radionuclides that emit alpha waves, such as ^225^Ac, ^213^Bi, and ^212^Pb, as opposed to conventional radiotherapy, which employs beta or gamma radiation. Due to their high LET, these alpha particles have short path lengths within tissues, allowing them to concentrate their radiation dose over a limited area. This precision makes TAT particularly well‐suited for targeting micrometastases and disseminated malignancies, reducing off‐target effects.^46^ TAT has shown promising results in clinical trials for various cancer types, including solid tumors, lymphomas, and leukemia. For example, by targeting prostate‐specific membrane antigen (PSMA), alpha‐emitting radioimmunoconjugates such as Ac‐225‐PSMA‐617 have demonstrated success in treating metastatic castration‐resistant prostate cancer.^47^

## 6 MANAGEMENT OF RADIOPHARMACEUTICAL WASTE

Proper management of radiopharmaceutical waste is essential for ensuring regulatory compliance and safety in RPT. Radiopharmaceutical waste should be stored in shielded containers, separated according to its half‐life, and allowed to decay to safe levels before being disposed of safely. While long‐half‐life isotopes require particular disposal techniques, short‐half‐life isotopes can be stored until they reach background radiation levels.^48^ After treatment, patient excreta (sweat, feces, and urine) may contain residual radioactivity; therefore, it is important to collect, categorize, and dispose of them separately to avoid accidental exposure. Depending on the isotope, this waste may be incinerated or disposed of using conventional sewage systems after its radioactivity has decreased to acceptable levels. Liquid radioactive waste might need to be stored for decay or chemically treated, including contaminated washings.^49^

## 7 SAFETY IN RADIOPHARMACEUTICALS

In nuclear medicine, ensuring the safety and effectiveness of radiopharmaceuticals is paramount. Before being made widely available, a new medicine must undergo extensive evaluation that provides detailed information about its safety and effectiveness. Once a new compound is developed, methods are established for its description, identification, and determination.^50^, ^51^ Clinical trial phases 1–3 are conducted after developing a stable pharmaceutical form and establishing a production system. Following these initial three phases of the trial, phase 4 involves ongoing surveillance by the manufacturer and registration authorities to ensure continued safety and effectiveness.^52^, ^53^

Before selecting safety indicators, it is crucial to consider the primary concerns to be monitored, managed, and modified. The purpose of the safety performance indicators is to inform stakeholders about safety, motivate them to prioritize it, and support changes that enhance safety. When selecting the indicators, it is essential to focus on what needs to be observed rather than how monitoring will be done. Otherwise, the selection of indicators could be distorted by focusing on what is considered feasible or practical to measure rather than focusing on the data needed to assess the organization's degree of safety.^54^, ^55^

## 8 CHALLENGES IN RADIOPHARMACEUTICALS

Radiopharmaceuticals are drugs that can be injected, inhaled, or swallowed, delivering DNA‐damaging radioactivity to cancer cells in tumors or circulating in the bloodstream in the form of alpha, beta, or conversion electrons.^56^ These medications are either “conjugated” (they have a structure of ligand‐chelator‐payload) or “neat” (they have a structure of payload or chelator‐payload only). They are composed of a radioactive payload and a radiochemical chelator, which are its two constituent parts, and each element poses potential risks to patient safety. To address these risks, the Cancer Therapy Evaluation Program has adopted a method for developing radiopharmaceuticals that considers payload, chelator, and potential ligand toxicity before starting any clinical trials.^57^ Radiopharmaceutical specificity evaluation using target antigen immunohistochemistry or micro autoradiography is a preliminary step in this strategy.^58^ Radiopharmaceuticals offer an effective treatment option when other conventional therapy modalities have failed. However, it is notable that despite their potential, radiopharmaceuticals are yet a standard component of cancer management, even though other “targeted therapies” with high clinical trial failure rates of 97% are widely adopted. In addition, the development and use of radiopharmaceuticals for therapy and imaging face challenges, including public fear of radioactivity and the perceived complexity of the treatment.^59^, ^60^

## 9 CONCLUSION

The advent of advanced radiopharmaceuticals has significantly enriched the landscape of cancer therapy, offering more effective, targeted, and personalized treatment strategies. The precision of radiopharmaceuticals to target the tumor microenvironment exemplifies the cutting‐edge of minimally invasive treatment. RPT has demonstrated high efficacy with minimal toxicity compared to other systemic cancer treatment options. The ability to select different radioligands that uniquely target molecular receptors or intracellular components makes them suitable for personal patient‐tailored therapy in modern cancer management. The integration of diagnostic imaging and therapeutic intervention, known as theranostics, heralds a new era for RPT. The potential for combination therapies, integrating radiopharmaceuticals with immunotherapy or chemotherapy, presents new opportunities for synergistic effects that could improve efficacy and patient outcomes. However, novel particle drug delivery systems, including the use of NPs, continue to enhance targeted therapy efficacy and safety. The increasing number of successful studies exploring new drug delivery agents and different delivery systems of radionuclide particles could increase the effectiveness and expand the applicability of these treatments. Regulatory agencies such as the FDA, EMA, and IAEA are pivotal in ensuring radiopharmaceuticals meet rigorous quality standards. Adherence to GMP is essential to protect public health, preserve the manufacturer's reputation, and enhance confidence in RPT. Despite these measures, maintaining the stability and sterility of these sensitive products remains a complex challenge, yet it is critical for their safe and effective use. The novel dynamic intersection between scientific ingenuity and clinical application of radiopharmaceuticals remains largely unexplored. Effective collaboration among multidisciplinary teams, including oncologists, nuclear medicine specialists, pharmacists, and researchers, is essential for translating these scientific breakthroughs into tangible patient benefits.

## CONFLICT OF INTEREST STATEMENT

The authors declare no conflicts of interest.

## ETHICS STATEMENT

Not applicable.

## Data Availability

No further data available.
